# Combinatorial therapy with resveratrol sensitizes glioblastoma to NKG2D CAR-T cells

**DOI:** 10.3389/fimmu.2026.1831927

**Published:** 2026-07-06

**Authors:** Wei Liang, Yexiao Tang, Muhammad Auwal Saliu, Mansur Dabai Salisu, Cuimei Chen, Zhiming Xu, Shu Xu, Maoxuan Liu, Xiaochun Wan

**Affiliations:** 1 Guangdong Immune Cell Therapy Engineering and Technology Research Center, Center for Protein and Cell-based Drugs, Institute of Biomedicine and Biotechnology, Shenzhen Institutes of Advanced Technology, Chinese Academy of Sciences, Shenzhen, China; 2 University of Chinese Academy of Sciences, Beijing, China; 3 Cancer Center, Shenzhen University of Advanced Technology General Hospital, Shenzhen, Guangdong, China; 4 School of Public Health, Xiangnan University, Chenzhou, Hunan, China

**Keywords:** car-t, combination therapy, glioblastoma, NKG2D, resveratrol

## Abstract

Chimeric antigen receptor T-cell (CAR-T) therapies have shown potential in clinical trials for glioblastoma, yet treatment responses vary due to heterogeneous antigen expression and post-treatment immune escape. NKG2D−based CAR-T cells have exhibited a favorable safety profile in patients with hematologic malignancies and demonstrated potent antitumor activity in xenograft models, including those of glioblastoma. Nevertheless, glioma cells could evade immune recognition by downregulating or proteolytically shedding NKG2D ligands. To enhance the efficacy of NKG2D CAR-T therapy, we investigated its combination with preclinical agents capable of penetrating the blood-brain barrier that could upregulate NKG2D ligands on glioma cells. Our study revealed that resveratrol (RSV), a bioactive polyphenol, significantly increased the surface expression of NKG2D ligands on glioblastoma cells. RSV pretreatment sensitized these cells to NKG2D CAR-T-mediated killing *in vitro*. Additionally, the combination of RSV with NKG2D CAR-T cells demonstrated potent antitumor activity *in vivo.* Mechanistically, RSV potentially induced NKG2D ligand expression via activation of the p53 signaling pathway. These preclinical findings identify RSV as a promising pharmacological adjuvant that enhances NKG2D CAR-T efficacy in glioblastoma, supporting further translational and clinical evaluation of this combinatory approach.

## 1 Introduction

Glioblastoma represents the most common primary malignant tumor of the central nervous system, characterized by its highly invasive nature, frequent recurrence, and formidable therapeutic challenges. Current standard clinical management involves surgical resection followed by radiotherapy or chemotherapy (1). These conventional approaches have resulted in a dismal 5-year survival rate of less than 10% and a median overall survival of under 2 years (2, 3). Despite immunotherapeutic strategies, including peptide-based vaccines (4, 5) and immune checkpoint inhibitors (6, 7), have demonstrated promising preliminary outcomes in glioblastoma studies, the majority of clinical trials targeting glioblastoma have yielded unsatisfactory results. Therefore, the development of novel immunotherapeutic interventions for glioblastoma remains an urgent unmet medical need.

Adoptive cellular therapy utilizing chimeric antigen receptor (CAR) T-cells has demonstrated significant clinical efficacy in treating hematologic malignancies. Notably, two CAR-T cell products currently approved by the U.S. FDA exhibit robust therapeutic responses against B-cell acute lymphoblastic leukemia (B-ALL) and diffuse large B-cell lymphoma (DLBCL) (8). This success has generated substantial optimism for extending CAR-T therapy to solid tumors, particularly glioblastoma. Early-phase clinical investigations have evaluated CAR constructs targeting EGFR and IL13Rα2 in recurrent glioblastoma (NCT02208362, Phase I) (9). Despite promising initial outcomes, these glioblastoma-directed CAR-T trials have demonstrated limited antitumor activity due to heterogeneous target antigen expression and tumor immune escape mechanisms (10, 11). Thus, overcoming target antigen downregulation or loss to prevent immune evasion represents a critical challenge for enhancing the efficacy of CAR-T therapies against solid tumors.

NKG2D, an activating immune receptor, plays a crucial role in innate and adoptive immune responses, which is mainly expressed on NK cells and CD8^+^ T cells (12, 13). To date, human NKG2D recognizes eight NKG2D ligands (NKG2DLs), including MHC I Chain-related molecules A and B (MICA and MICB) and six cytomegalovirus UL16-binding proteins (ULBP1-6) (14, 15). NKG2D ligands are generally absent on the surface of healthy cells while this inducible expression is markedly enhanced by hematologic malignancies and solid tumors (16). Recent evidence has highlighted that NKG2D CAR-T targeting NKG2DL exhibits favorable therapeutic efficacy in the treatment of solid tumors, particularly gliomas (17), implying that NKG2DL is a potential target for CAR-T therapy. For instance, NKG2D CAR-T(KD-025) in the treatment of relapsed or refractory NKG2DL^+^ solid tumors (NCT04550663, Phase I). However, malignant glioma cells frequently evade NKG2D-mediated immune surveillance via proteolytic shedding or exosome-mediated secretion of ligands, generating soluble NKG2D ligands that impair immune recognition (18, 19). The density of target antigens on tumor cells is crucial for the cytotoxic effectiveness of CAR-T cell therapy (20). Thus, upregulating NKG2D ligand expression pharmacologically represents a critical strategy for improving the efficacy of NKG2D-targeted CAR-T therapy in glioblastoma.

Resveratrol (trans-3,4,5-trihydroxystilbene; RSV), a bioactive polyphenolic compound, exhibits high blood-brain barrier permeability and low neurotoxicity. Its broad therapeutic profile, including cardioprotective, antioxidant, anti-inflammatory, and anticancer properties, underpins extensive research interest (21). Crucially, RSV confers cardiovascular protection through coordinated modulation of oxidative stress, ferroptosis inhibition, endothelial function preservation, and lipid metabolism regulation (22). Previous studies have shown that RSV-mediated blockade of Notch/NF-κB and HMGB1/NF-κB signaling pathways attenuates age-associated cardiac damage and monocyte survival-driven inflammatory myocardial injury, respectively (23, 24). Additionally, the combination of RSV and curcumin significantly inhibits the NF-κB signaling pathway, exerting a potent synergistic anti-inflammatory effect and concomitantly reducing vascular inflammation (25). Recent studies have demonstrated that RSV reduces the growth of solid tumors, including liver cancer, breast, pancreas, colon, prostate cancer and malignant glioblastoma (26–28). Excitingly, combination RSV with temozolomide down-regulate the the expression of MGMT and STAT3/Bcl-2 signaling pathway, which further induces the apoptosis of glioma cells (22). However, it remains unclear whether RSV enhances CAR-T cell sensitivity to glioma cells and improves their therapeutic efficacy. The underlying regulatory mechanisms also require further elucidation.

In this study, we investigated the adjuvant effect of resveratrol (RSV) on NKG2D CAR-T cell therapy for glioblastoma and its mechanism via RNA-Seq. RSV was found to upregulate NKG2D ligand expression on tumor cells, enhancing CAR-T cytotoxicity *in vitro*. The combination therapy demonstrated potent anti-tumor efficacy with minimal toxicity *in vivo*. Collectively, our results reveal a novel therapeutic paradigm, positioning RSV as a promising combinatory agent to advance CAR-T immunotherapy for solid tumors.

## 2 Materials and methods

### 2.1 Cell culture

U251 and A172 cells were obtain from the Chinese Academy of Sciences’Cell Bank/Stem Cell Bank. U87 cells were provided by the American Type Culture Collection (ATCC, USA). All the cancer cells were cultured in DMEM (10% FBS and 1% penicillin/streptomycin) at 37 °C with 5% CO2 for indicated time for assays.

### 2.2 Plasmid construction and lentiviral package

The lentiviral plasmid of pWPXLD- NKG2D CAR was constructed according to our previous study (29). Subsequently, according to CytoPEI TM Transfection Reagent’s manufacturer protocols, HEK 293T cells were transfected with the lentiviral plasmid and the packaging plasmids at a ratio of 5:3:2, such as psPAX2 and pMD2.G. At 48 h after transfection, the supernatants were collected and concentrated with PEG 8000 in an appropriate proportion.

### 2.3 Generation of CAR-T cells

The human primary T cells were transfected with the supernatants of NKG2D CAR-expressing lentiviral vectors as previously describe (29). Briefly, after isolation from the peripheral blood mononuclear cells (PBMC) of healthy donors, T cells were activated with Dynabeads ™ CD3/CD28 (Thermo Fisher Scientific) at a 1:1 ratio and cultured in RPMI-1640 (10% FBS and 1% penicillin/streptomycin) with IL-2 (50 U/mL, Novoprotein) at 37 °C for 24 h. Activated T cells were transfected with the supernatants of lentiviral vectors, followed by examination the percentage of NKG2D^+^ cells using flow cytometry. All the studies involving animal subject were approved by the Institutional Review Board of the Shenzhen Institute of Advanced Technology, Chinese Academy of Sciences. Written informed consent was obtained from all participants prior to their involvement, and all procedures strictly adhered to international ethical guidelines governing biomedical research involving human subjects.

### 2.4 Flow cytometry

Cells were collected and washed twice with PBS, followed by resuspended with PBS containing 2% FBS. Subsequently, the samples were stained with labeled primary antibodies on ice for 30 min and washed twice with PBS before flow cytometry assay. The labeled primary antibodies as follows: APC anti-human CD314 (NKG2D) antibody (Cat# 320807), Anti-human ULBP1 antibody (Cat# MAB1380-SP), anti-human ULBP2/5/6 antibody (Cat# MAB1298), anti-human ULBP3 antibody (Cat# MAB1517), Anti-human ULBP4 antibody (Cat# sc-53133), APC Anti-human IgG Fc recombinant antibody (Cat# 366905) and APC anti-human MICA/B antibody (Cat# 320907). For intracellular cytokines staining, NKG2D CAR-T cells were co-cultured with U251 cells treated with Brefeldin A (BD Biosciences) at indicated ratios for 6 h. T cells were stained with Zombie Aqua Live/dead, APC/Cyanine 7 CD45 and PerCP/PC5.5 CD3, followed by fixation and permeabilization. BV421 IL-2, APC TNF-α and PE IFN-γ antibody were used for intracellular staining as described above before was examined by flow cytometry and analyzed by FlowJo software.

### 2.5 Immunofluorescence assay

U251 cells were deposited onto slides using a Cytospin 4 centrifuge (Thermo Scientific; 1,000 ×g, 3 min) and fixed with methanol for 5 min. After blocking with 1% bovine serum albumin (BSA) for 1 h at 37 °C, slides were washed twice with PBST and once with PBS. Cells were then incubated with an anti-human ULBP2 antibody (Cusabio, 1:200 dilution) for 1 h at 37 °C, followed by an Alexa Fluor 488−conjugated goat anti−rabbit secondary antibody (Abcam; 1:800 dilution) under the same conditions. Following three washes, nuclei were counterstained with DAPI (Beyotime). Slides were mounted and imaged using a Zeiss ApoTome microscope. For immunofluorescence assay from tumor, tumor tissues were fixed in 4% paraformaldehyde, paraffin-embedded, and sectioned (4 μm). Sections were dewaxed, rehydrated, and subjected to heat-induced antigen retrieval. After blocking with 5% BSA for 30 min, sections were incubated overnight at 4 °C with anti-CD45 and anti-CD3 primary antibodies. Following washes, sections were incubated with fluorophore-conjugated secondary antibodies (1 h, room temperature, dark). Images were acquired using a fluorescence microscope. Fluorescence images were analyzed using Saiviewer software (Servicebio, Wuhan, China). Statistical significance was assessed by two-tailed Student’s t-test.

### 2.6 Treatment of glioblastoma cell lines with RSV

Glioblastoma cells were seeded at a density of 5,000 cells in 96-well plates. After 24 h attachment, different concentrations of RSV (0, 3.125,6.25, 12.5,25,100 μM) were added. Cell viability was detected using a CCK-8 kit (Shanghai Yeasen Biotech) at 48 h of treatment according to manufacturer. For flow cytometric assay, the cells were seeded at a density of 10,000 cells/mL in 24-well plates. Upon attachment, the cells were incubated with 20 μM RSV for 48 h. Followingly, the cells were harvested to stain with NKG2D ligands and examined by flow cytometry. For ELISA assay, the cells were seeded at a density of 20,000 cells/mL in 12-well plates. Upon attachment, the cells were incubated with 20 μM RSV for 48 h. Followingly, the supernatants were harvested to detect ULBP2 expression using a Human ULBP2 ELISA Kit (Shanghai Aimeng Youning Biotechnology).

### 2.7 Cytotoxicity assay

Cytotoxic activity of *in vitro* was assessed using the xCELLigence RTCA SP instrument (ACEA Biosciences) with minor modification. Glioblastoma cells were seeded at a density of 5,000 cells in Corning^®^ spheroid 96-well microplates. Following a 24 h attachment period, cells were incubated with 20 μM RSV for 12 h and subsequently washed with PBS. The medium was then replenished with RPMI-1640 (supplemented with 10% FBS and 1% penicillin/streptomycin) containing either NKG2D CAR-T cells or untransduced (UTD) cells at specified effector-to-target (E:T) ratios. All co-culture conditions were established in triplicate. Cytotoxic activity, quantified by monitoring the viability of adherent target cells for at least 24 h, was measured through real-time cell index (CI) values. CI normalization was performed at the experimental endpoint using RTCA Software Pro (v2.3.0) to minimize well-to-well variation. Final percentage cytotoxicity was calculated as follows:


% cytotoxicity = ((CI no effector−CI effector)/(CI no effector)) × 100

### 2.8 Glioblastoma xenograft mouse model *in vivo*

All mouse experiments were conducted in compliance with relevant institutional guidelines and regulatory standards, with ethical approval granted by the Institutional Animal Care and Use Committee of the Shenzhen Institute of Advanced Technology, Chinese Academy of Sciences. Female B-NDG mice (6 weeks old; Biocytogen, Beijing) were maintained under specific-pathogen-free (SPF) conditions with a 12 h light-dark cycle and subjected to regular health monitoring. B-NDG mice were received subcutaneous inoculations of 2 × 10^5^ U251 tumor cells. Tumor growth was measured every 3 days. The mice were intravenously injected with RSV (5 mg/kg) or vehicle control at day 14, 16 and 18. Tumor-bearing mice received intravenous injections of either NKG2D CAR-T cells or untransduced T (UTD) cells at a dose of 2 × 10^5^ cells (high dose) or 0.5 × 10^5^ cells (low dose) per mouse when volumes reached approximately 100 mm³ (calculated as volume = width² × length/2). Throughout the study, tumor volumes and murine body weights were serially monitored. At the experimental endpoint (Day 45), mice were deeply anesthetized with 3% isoflurane and humanely euthanized by cervical dislocation. Tumor tissues and major organs (heart, liver, spleen, lungs, and kidneys) were then collected for subsequent analysis.

### 2.9 Histological studies

Specimens from heart, liver, spleen, lungs and kidney were immersion-fixed in 4% paraformaldehyde (PFA; Beyotime Biotechnology, Shanghai) for 24 h. Fixed tissues underwent sequential dehydration through an ethanol gradient, xylene clearance, and paraffin embedding. Sectioning was performed at 4 μm thickness followed by standard haematoxylin and eosin (H&E) staining. Histological evaluation was conducted using an Olympus BX53 light microscope, with image acquisition and morphometric analysis performed using AxioVision SE64 software (v4.9.1, Zeiss).

### 2.10 Orthotopic intracranial glioblastoma model

A single-cell suspension of vigorously growing U251 human glioma cells was adjusted to a density of 2 × 10^4^ cells/μL, mixed on ice with an equal volume of Matrigel, and kept in an ice-water bath until use. The interval from cell harvest to complete intracranial implantation was strictly limited to within 90 minutes. Female B-NDG mice (6–8 weeks old, SPF grade) were acclimated in the animal facility for one week, then anaesthetized with inhaled isoflurane. Following scalp depilation and disinfection, each mouse was secured in a stereotaxic frame. A midline longitudinal incision was made to expose the bregma, and a burr hole was drilled 1 mm anterior and 2 mm lateral (left) to the bregma. A micropipette loaded with 5 μL of the cell suspension was mounted on the stereotaxic arm and advanced vertically to a depth of approximately 3 mm. The suspension was infused at a constant rate of 2.5 μL/min, after which the needle was left in place for 2 min to prevent backflow along the needle tract. Upon needle withdrawal, the cranial defect was sealed with dental cement. The scalp incision was sutured following standard disinfection, and the surgical site was re-disinfected post-operatively.

### 2.11 Bioluminescence imaging *in vivo*

A working solution of D−luciferin potassium salt (15 mg/mL) was prepared in sterile DPBS and filter−sterilized through a 0.2 μm membrane. The solution was used immediately, or aliquoted and stored at -80 °C, avoiding repeated freeze–thaw cycles. Prior to use, aliquots were thawed at 4 °C and kept chilled and protected from light. The working solution was administered via intraperitoneal injection at a volume of 10 μL per gram of body weight. Imaging was performed 10-20 min post−injection, during which the mouse was maintained under isoflurane anesthesia. Once the bioluminescent signal reached a stable plateau, the mouse was placed on the stage of a small−animal *in vivo* imaging system (IVIS, PerkinElmer) for image acquisition. The total fluorescence intensity was calculated using ImageJ.

### 2.12 Cytokine release assays

Cytokine release assays of serum from the co-culture assay or U251 cells bearing-orthotopic mouse model, the cytokine levels were examined by Mult-analyte Flow assay (BD Biosciences) according to the manufacturer’s instructions. Data were analyzed by FlowJo software.

### 2.13 RNA-Seq assay

U251 cells (2 × 10^5^) were treated with 20 μM RSV or vehicle control for 48 h, with four sample replicates (n=4). Total RNA was isolated from harvested cells using TRIzol™ reagent (Invitrogen) according to the manufacturer’s protocol, and subjected to RNA-Seq at Genedenovo (Guangzhou, China). mRNA was enriched using oligo(dT)-coated magnetic beads and fragmented by ultrasound. First-strand cDNA was synthesized with random primers and M-MuLV reverse transcriptase, followed by RNaseH treatment and second-strand synthesis with DNA polymerase I. After end repair, A-tailing, and adapter ligation, 200-bp fragments were selected with AMPure XP beads, PCR-amplified, and purified to obtain the final library. Reads were mapped to the human genome (Ensembl v100) using HISAT2 (v2.2.4). RNA-seq data were analyzed on the OmicSmart platform (http://www.omicsmart.com) with DESeq2 (group comparisons) or edgeR (pairwise comparisons), followed by Ontology (GO) enrichment, KEGG pathway enrichment and GSEA analysis (30).

### 2.14 Quantitative real-time RT-PCR

Total RNA was isolated using TRIzol, treated with DNase, and reverse-transcribed into cDNA (Novoprotein kit). Quantitative PCR was performed in triplicate on a Bio-Rad CFX Connect system using SYBR Green SuperMix (NovoStart). Normalization was conducted against GAPDH, and relative expression levels were calculated using the 2^−ΔΔCT^ method. All primer sequences are listed in **Supplementary Table 1**.

### 2.15 Molecular docking

Candidate targets identified through network pharmacology and enrichment analyses were subjected to molecular docking. The three−dimensional (3D) structures of the target proteins were retrieved from the Protein Data Bank (PDB; https://www.rcsb.org/), while the 3D structural files of the small−molecule compounds were obtained from PubChem. Potential binding sites on the target proteins were predicted, and the docking grid was defined using AutoDockTools. After parameter configuration, a docking control file specific to AutoDock Vina was generated. For each ligand-receptor pair, 100 independent docking runs were performed with AutoDock Vina. The conformation with the most favorable binding affinity was retained and saved in PDB format. Finally, ligand-receptor interaction patterns, including hydrogen bonds and hydrophobic contacts, were visualized and analyzed using PyMOL.

### 2.16 Western blot

U251 cells and A172 cells were lysed on ice for 30 min using RIPA buffer supplemented with 1% phosphatase inhibitor cocktail and 1% protease inhibitor cocktail. Lysates were centrifuged at 4 °C for 10 min (10, 000 rpm), and the supernatants were collected. Protein samples were mixed with SDS-loading buffer, boiled at 100 °C for 10 min, and separated by SDS-polyacrylamide gel electrophoresis (SDS-PAGE). After transfer onto a nitrocellulose membrane, the membrane was blocked with 1% bovine serum albumin (BSA) for 1 h at room temperature and washed three times with PBST (PBS containing 0.1% Tween-20). The membrane was then incubated overnight at 4 °C with the following primary antibodies (1:1,000 dilution): anti-phospho-p53 (Ser376) rabbit monoclonal antibody (Beyotime, AF1699), Caspase 3 Rabbit Monoclonal Antibody (Beyotime, AF1213) and anti-β-actin rabbit monoclonal antibody (Cell Signaling Technology, #8457). After washing with PBST, the membrane was incubated for 1 h at room temperature with horseradish peroxidase (HRP)-conjugated goat anti-rabbit IgG (Beyotime; 1:3,000 dilution), followed by three washes with PBST. Protein signals were detected using Super ECL Detection Reagent (YEASEN) and visualized with an Odyssey CLx imaging system (Image Studio).

### 2.17 Statistical analysis

The results are presented as the mean ± SD. Normality was assessed using the Shapiro-Wilk test, and homogeneity of variances was evaluated with the Levene test. For comparisons between two groups, a two-tailed t-test was applied when normality was not rejected, while one-way ANOVA with LSD, Duncan and Tukey’s multiple-comparisons test was used for comparisons for multiple groups. When normality was rejected, the Mann-Whitney test was used for two-group comparisons, and the Kruskal-Wallis test for multiple groups comparisons. Significance thresholds were defined as follows: *, *p* < 0.05; **, *p* < 0.01; ***, *p* < 0.001.

## 3 Results

### 3.1 NKG2DLs are expressed in glioblastoma

**Figure 1:**
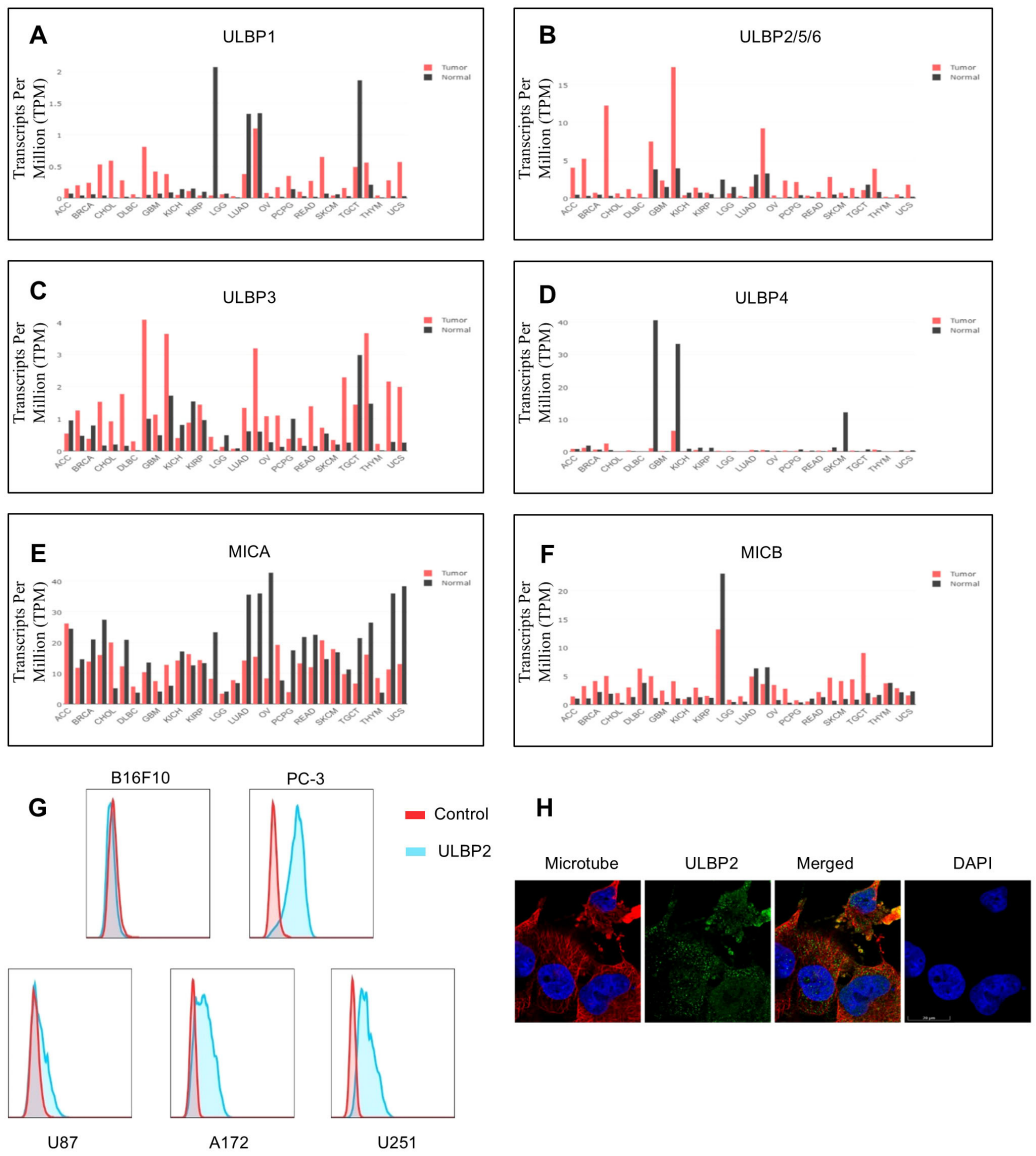
NKG2D ligands exhibit higher expression levels in glioblastoma. **(A-D)** Gene expression profiles of ULBP family proteins in tumor samples and matched normal tissues from GEPIA database. **(E, F)** Gene expression profiles of MIC family proteins in tumor samples and matched normal tissues from GEPIA database. **(G)** NKG2DLs expression were determined by Flow cytometry (n=3). **(H)** The ULBP2 expression was observed in U251 cells by immunofluorescence staining (n=3). Experiments were repeated a minimum of three times with consistent results.

According to GEPIA database, the mRNA levels of six different NKG2DLs were expressed in different types of tumors, including glioblastoma (**Figures 1A–F**). Consistent with these results, the levels of NKG2DLs were examined on three representative glioblastoma cell lines (U87, A172, U251) (**Figure 1G**). Meanwhile, the expression levels of ULBP2 were also found on PC-3 cells, except for B16F10 cells (**Figure 1G**). The ULBP2 expression in U251 cells was further observed by immunofluorescence staining (**Figure 1H**). The confirmed expression of NKG2DLs on glioblastoma cells provides a mechanistic rationale for targeting this malignancy with NKG2D CAR-T cell therapy. Based on this rationale, we engineered a lentiviral vector encoding an NKG2D-targeted chimeric antigen receptor (CAR). The CAR construct incorporated the extracellular domain of NKG2D fused to a CD8α hinge/transmembrane region, coupled with 4-1BB co-stimulatory and CD3ζ signaling domains according to our previous study (29).

### 3.2 RSV elevated the expression of NKG2DLs on glioblastoma cells

**Figure 2:**
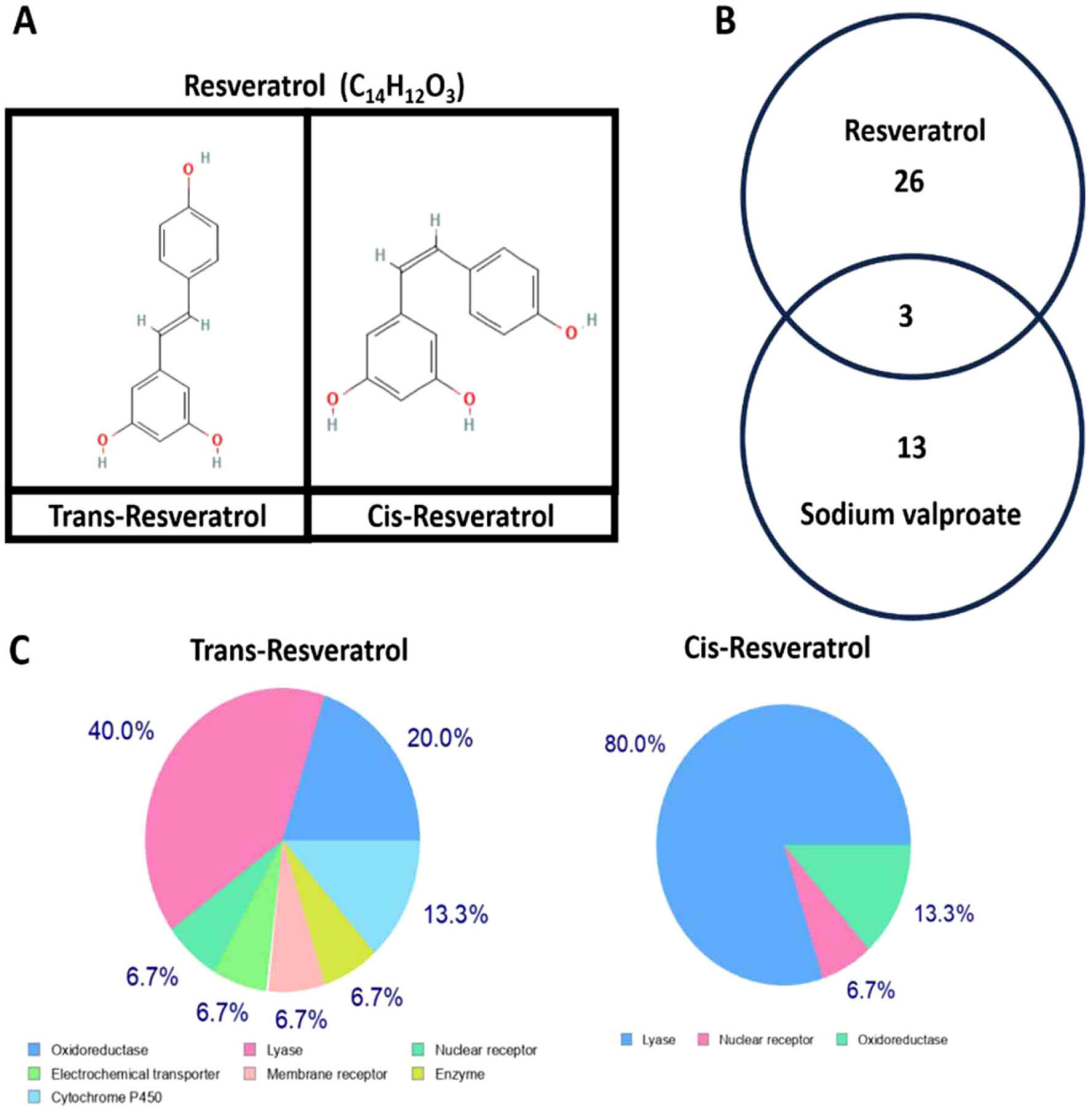
RSV structure and target prediction **(A)** Chemical structure of RSV. **(B, C)** RSV targets and its potential signaling pathways were predicted using DrugBank and Swisstarget databases.

RSV, a polyphenolic compound predominantly in the trans configuration with antioxidant and anti-inflammatory properties, was predicted by DrugBank database that revealed some common targets of Sodium valproate (**Figures 2A, B**). Additionally, the RSV targets were also predicted by Swisstarget database, including cell surface receptors and transcription factors (**Figure 2C**). We therefore hypothesized that RSV might enhance tumor immune recognition by upregulating NKG2DLs expression on glioblastoma cells, revealing a previously unknown immunoregulatory function.

**Figure 3:**
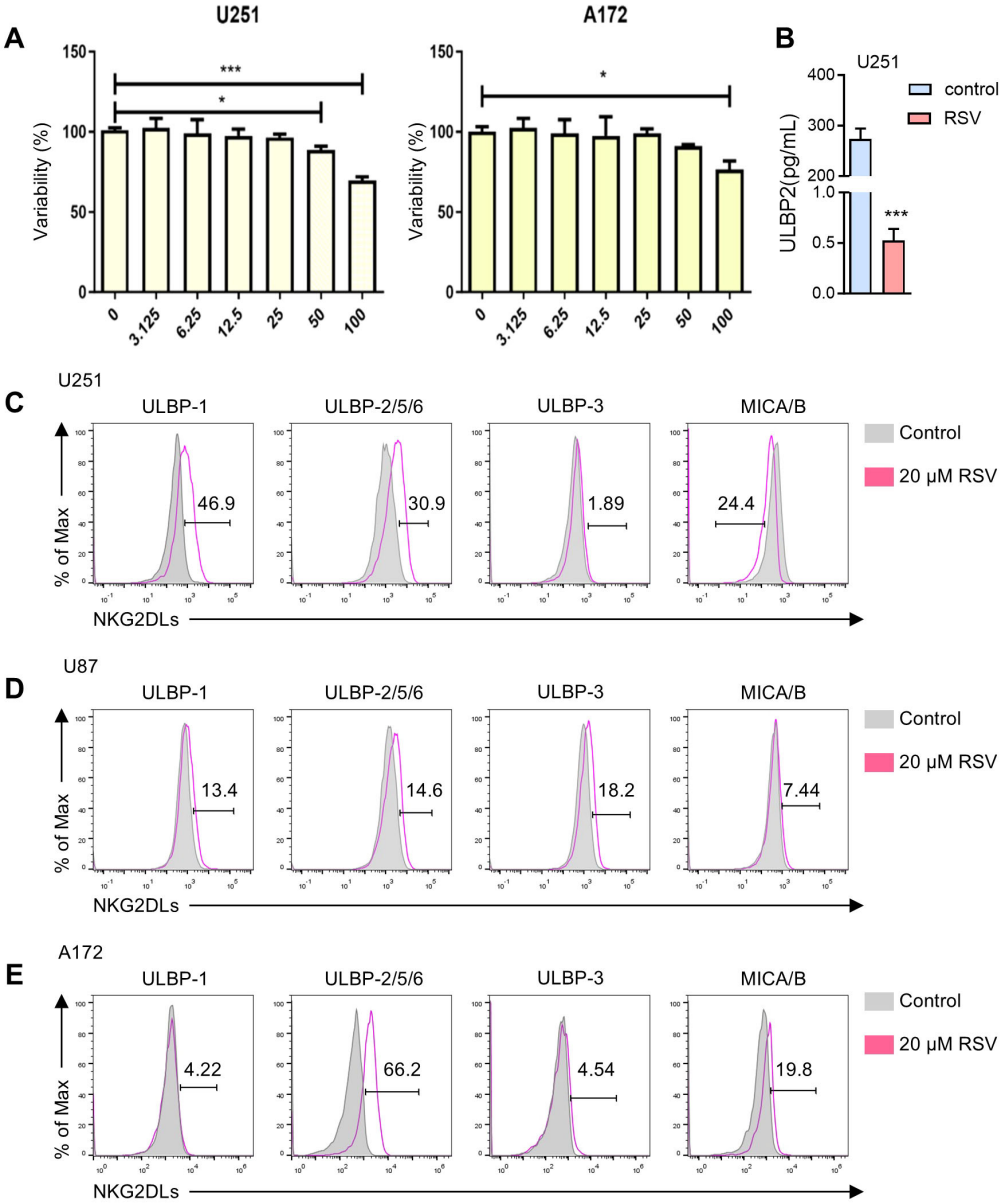
RSV treatment enhanced the expression levels of NKG2D ligands in glioblastoma. **(A)** The viability of U251 and A172 cells was detected by CCK-8 kits after treatment with different concentrations of RSV (n=3). **(B)** Soluble NKG2DLs in the supernatants of U251 cells were measured by ELISA after RSV treatment. (n=3). **(C-E)** NKG2DLs expression in glioblastoma cell lines were determined by Flow cytometry after RSV treatment (n=3). Experiments were repeated a minimum of three times with consistent results. *p < 0.05, ***p < 0.001, examined by a two-tailed t-test for two groups or one-way ANOVA for three or more groups.

Next, we investigated whether RSV upregulated expression of NKG2DLs on glioblastoma cells, thereby enhancing the sensitizing these tumors to lysis by NKG2D CAR-T cells. We first sought an *in vitro* RSV dose at which the majority of glioblastoma cells remained viable. The results indicated that between 12.5 to 25 μM RSV had lower negligible cytotoxicity on these cell lines, with > 95% cancer cell viability (**Figure 3A**). Consequently, we explored that the effect of 20 μM RSV treatment on expression of NKG2DLs on glioblastoma cells, including U251, U87 and A172 cells. RSV treatment significantly inhibited the proteolytic shedding of soluble NKG2DLs from U251 glioblastoma cells (**Figure 3B**). Notably, treatment with 20 μM RSV markedly induced the expression of ULBP2/5/6 on glioblastoma cells (**Figures 3C–E**), potentially enhancing their sensitivity to NKG2D CAR-T cells.

### 3.3 NKG2D CAR-T combined with RSV enhances cytotoxicity against glioblastoma cells

**Figure 4:**
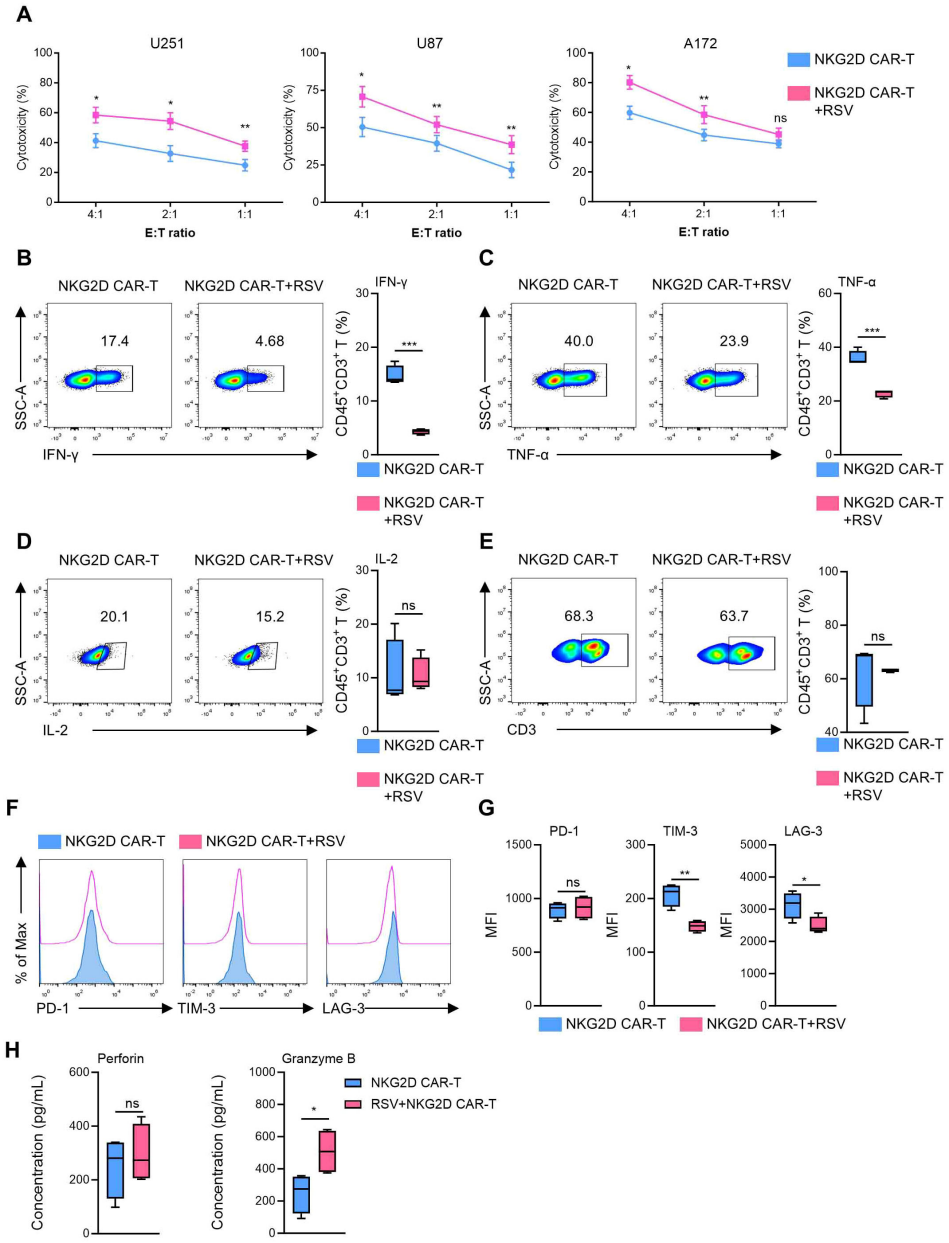
RSV treatment enhanced the cytotoxicity of NKG2D CAR-T cells against glioblastoma cells. **(A)** 20 μM RSV pretreatment elevated NKG2D CAR-T-mediated specific cytotoxicity against U251, U87 and A172 cells. The results are presented as the mean ± SD (n = 3). **(B-D)** After were pretreated with RSV for 3 h, U251 cells were co-cultured with NKG2D CAR-T cells for 6 h (n=4). Followingly, intracellular cytokines IFN-γ, TNF-α and IL-2 were detected by Flow cytometry. **(E)** After were pretreated with RSV for 3 h, U251 cells were co-cultured with NKG2D CAR-T cells for 24 h. Followingly, CD45^+^CD3^+^ CAR-T cells were examined by Flow cytometry (n=4). **(F-H)** After were pretreated with RSV for 3 h, U251 cells were co-cultured with NKG2D CAR-T cells for 24 h. Followingly, T-cell exhaustion markers PD-1, TIM-3 and LAG-3 were measured by Flow cytometry (n=4). Meanwhile, Perforin and granzyme B concentrations in the co-culture supernatant were determined by flow cytometry (n=4). Experiments were repeated a minimum of three times with consistent results. *p < 0.05, **p < 0.01, ***p < 0.001, examined by a two-tailed t-test.

Next, we further investigated that whether RSV treatment enhanced sensitivity of NKG2D CAR-T cells to glioblastoma cells. As expected, RSV combined with NKG2D CAR-T cells treatment significantly enhanced the cytotoxicity against glioblastoma cells compared with NKG2D CAR-T cells alone (**Figure 4A**), which further led to an obvious reduction in cytokine release by CAR-T cells, including IFN-γ and TNF-α, except IL-2 (**Figures 4B–D**). Consistent with the absence of significant changes in IL-2 secretion, CAR-T cell proliferation was likewise unaffected (**Figure 4E**). Notably, RSV treatment effectively reduced the CAR-T cells exhaustion, as revealed by the down-regulated expression of TIM-3 and LAG-3 (**Figures 4F, G**). Notably, RSV combined with NKG2D CAR-T cells treatment significantly enhanced the expression of Granzyme B (**Figure 4H**). These results suggest that RSV treatment increases NKG2DLs expression on glioblastoma cell surfaces, thereby enhancing NKG2D CAR-T cells cytotoxicity against tumor cells.

### 3.4 NKG2D CAR-T cells combined with RSV exhibit potent anti-tumor efficacy against ectopic glioblastoma model *in vivo*

**Figure 5:**
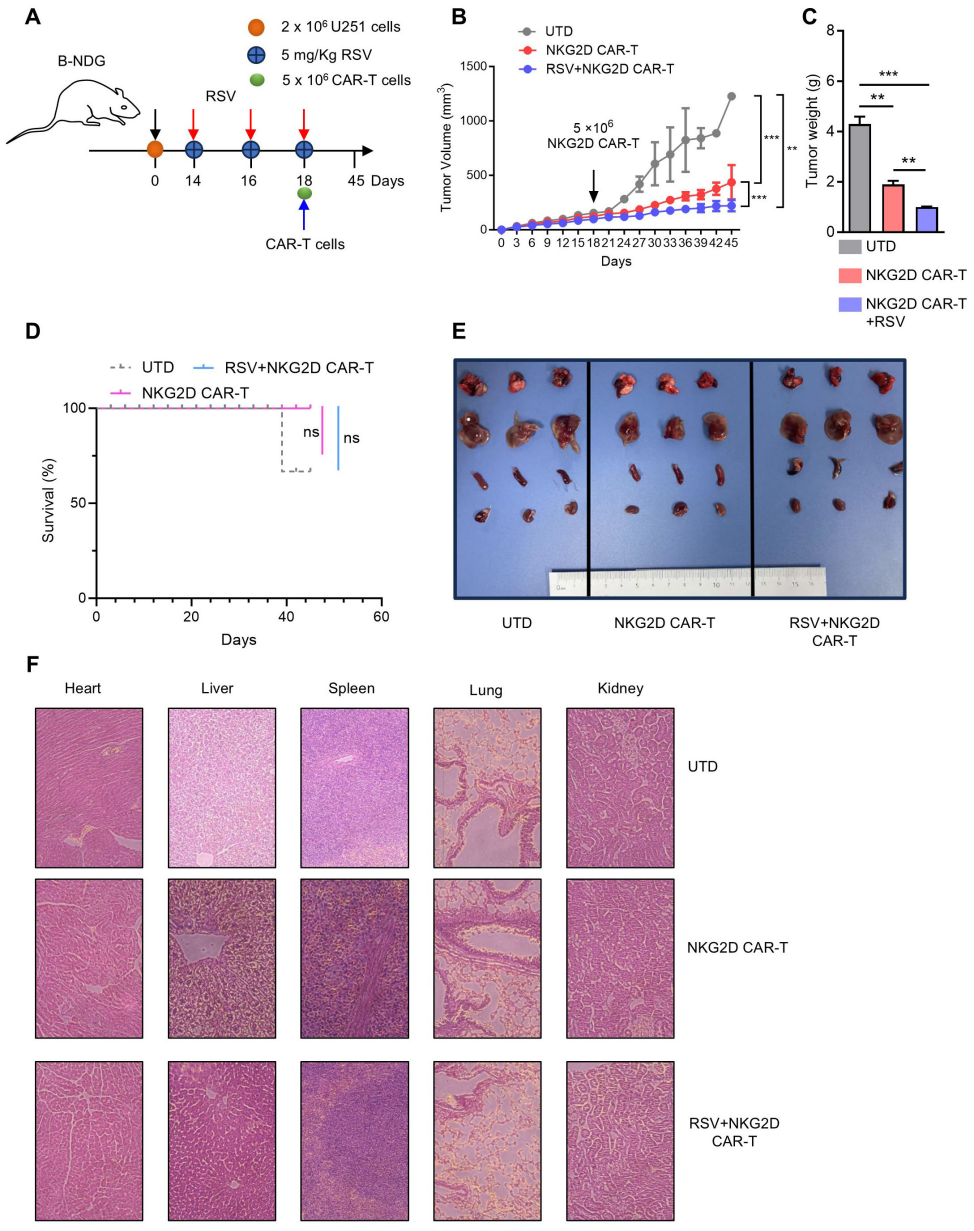
RSV combined with NKG2D CAR-T cells treatment effectively inhibited glioblastoma growth in a subcutaneous model. **(A)** Schematics of glioblastoma U251 cells model for combinational therapy (n=3 per group). **(B)** Tumor growth curves were recorded. **(C)** Tumor weight data were collected at the endpoint (27 days post T-cell infusion). **(D)** Kaplan-Meier survival curves of mice were recorded and analyzed by Log-rank test. **(E)** Critical organs were showed. **(F)** Critical organs were performed by H&E staining. Experiments were repeated a minimum of three times with consistent results. **p < 0.01, ***p < 0.001, examined by one-way ANOVA.

We next determined whether NKG2D CAR-T combined with RSV could inhibit glioblastoma growth *in vivo*. Using a glioblastoma subcutaneous model, we performed subcutaneous injections in B-NDG mice with RSV, followed by an infusion of either UTD cells or high dose NKG2D CAR-T cells (**Figure 5A**). Tumor growth was tracked by determination tumor volume and tumor weight. Our results indicated that UTD group failed to inhibit the growth of U251 tumors, while NKG2D CAR-T cells sharply reduced tumor burden (**Figures 5B, C**). Notably, NKG2D CAR-T cells combined with RSV further significantly reduced tumor burden compared with only NKG2D CAR-T cells groups (**Figures 5B, C**). Additionally, no prominent difference of survival was observed (**Figure 5D**). Meanwhile, no obvious abnormalities of major organs were observed, as revealed by H&E staining (**Figures 5E, F**), indicating the minimal toxicity of combinational therapy. Since potential side effects of CAR-T cells therapy, such as cytokine release syndrome, we explored whether the low dose of NKG2D CAR-T cells combined with RSV could suppress glioblastoma growth *in vivo*. The results showed that compared with only NKG2D CAR-T cells, low dose of NKG2D CAR-T cells combined with RSV significantly reduced tumor burden, revealed by tumor volume and tumor weight (**Supplementary Figures 1A–C**). In addition, no prominent difference of survival was observed (**Supplementary Figure 1D**). Collectively, these findings suggest that RSV enhances the anti-tumor activity of NKG2D CAR-T cells against glioblastoma.

### 3.5 The combination of NKG2D CAR−T cells and RSV exerts robust anti-tumor efficacy against orthotopic intracranial glioblastoma

**Figure 6:**
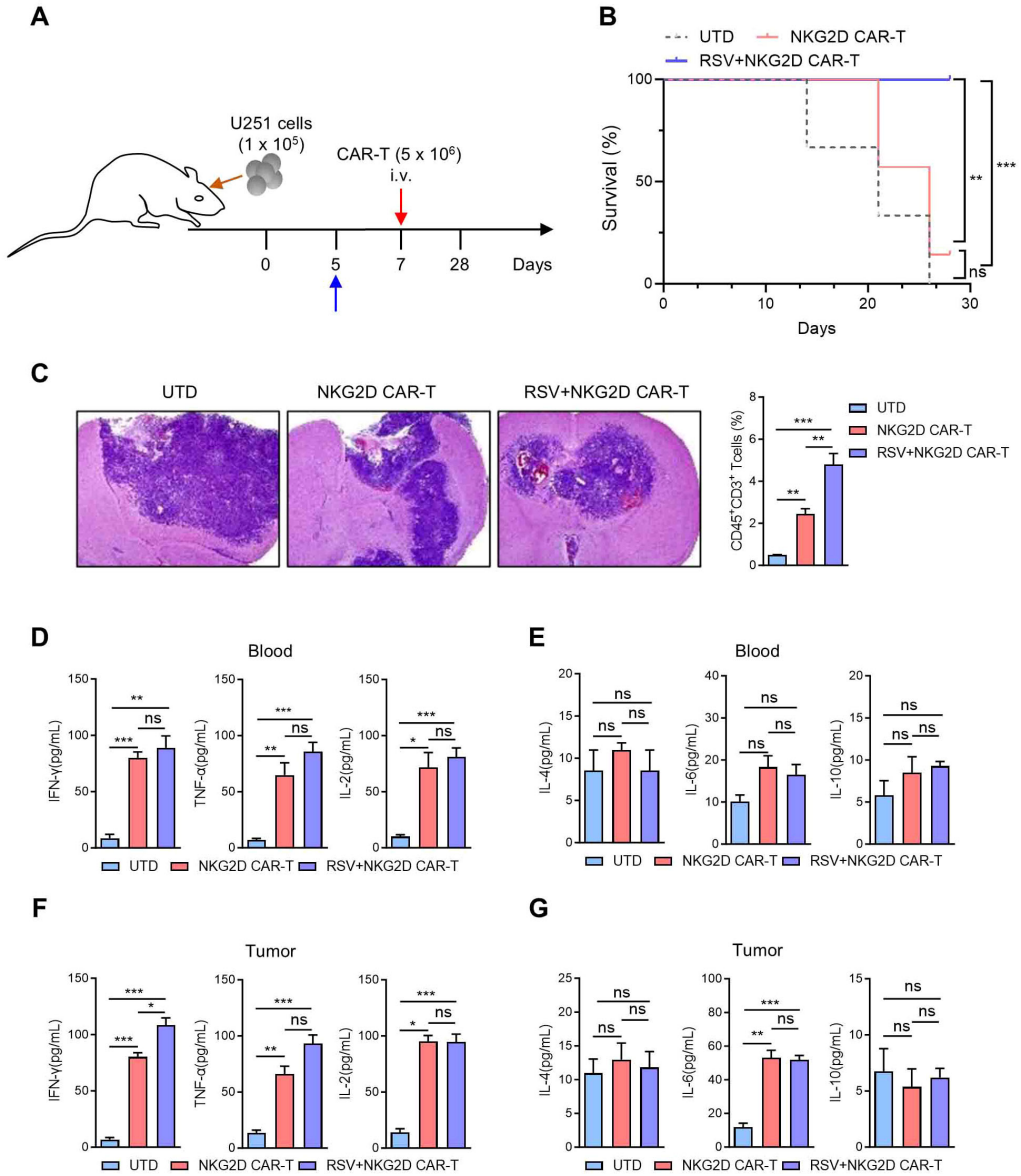
RSV combined with NKG2D CAR-T cells treatment exhibit potent anti-tumor efficacy against orthotopic intracranial glioblastoma. **(A)** Schematics of orthotopic intracranial glioblastoma for combinational therapy (n=9 per group). **(B)** Kaplan-Meier survival curves of mice were recorded and analyzed by Log-rank test. **(C)** Immunofluorescence staining was performed on the tumor tissues. **(D, E)** Cytokines levels from Peripheral blood were determined on days 14 after combination therapy. **(F, G)** Cytokines levels from tumor were determined at the endpoint. *p < 0.05, ***p < 0.001, ns: no significance, examined by one-way ANOVA.

Given that RSV crosses the blood-brain barrier, we established an orthotopic U251 glioma model (**Figure 6A**). Intracranial tumors progressed markedly faster than subcutaneous ones, and UTD groups succumbed within four weeks, presumably due to rapid tumor expansion causing intracranial hypertension. The results showed that combination therapy significantly enhanced the survival compared with or without NKG2D CAR-T cells treatment (**Figure 6B**). Notably, RSV synergized with NKG2D CAR-T cells treatment remarkably increased T−cell infiltration into the tumor (**Figure 6C**), indicating that RSV synergizes with CAR−T cells to potently suppress orthotopic U251 growth. The cytokines release from Peripheral blood or tumor was no significantly changed between NKG2D CAR-T cells with or without RSV treatment, except IFN-γ from tumor (**Figures 6D–G**). The very low concentrations of these cytokines suggest that the combination therapy exhibits minimal toxicity.

### 3.6 RSV enhances the expression of NKG2DLs on glioblastoma cells via p53 signaling pathway

**Figure 7:**
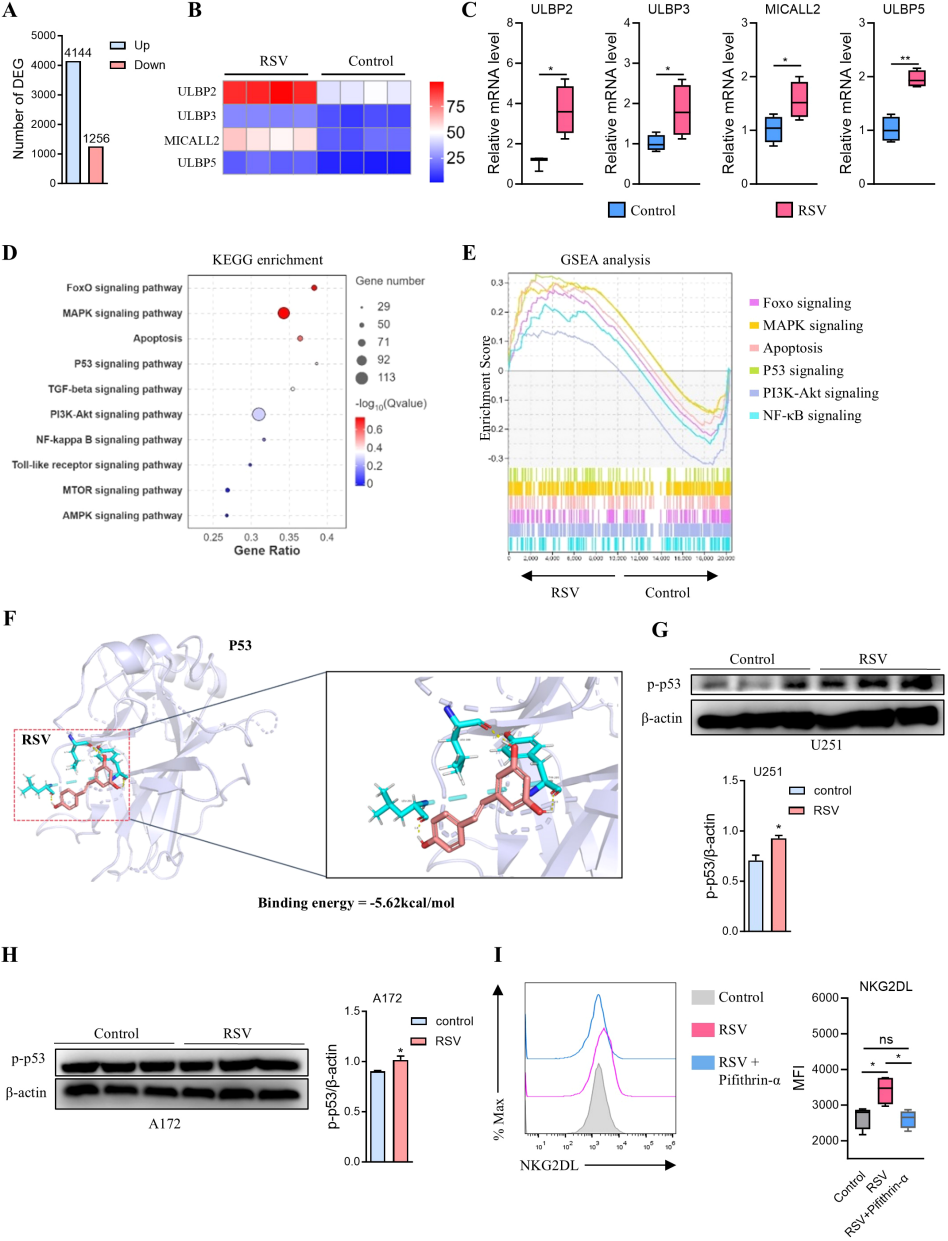
RSV enhanced NKG2D ligands expression in glioblastoma via p53 signaling. **(A)** Differentially expressed genes from U251 cells were identified after RSV treatment for 48 h (n=4). **(B, C)** Heatmap showed the mRNA expression of NKG2D ligands, as revealed by qPCR. **(D, E)** KEGG enrichment indicated that critical signaling pathway involved in RSV-induced the upregulation of NKG2D ligands, as uncovered by GSEA analysis. **(F)** Molecular docking predicted downstream protein signals bound to RSV. **(G, H)** Phosphorylated p53 signaling was detected by Western blot upon RSV treatment for 6 h (n=3). **(I)** After were pretreated with RSV for 3 h, U251 cells were treated with inhibitor (20 μM Pifithrin-α) of p53 signaling for 6 h. NKG2D ligands expression was measured by Flow cytometry (n=4). Western blot and Flow cytometry were repeated for three times. *p < 0.05, **p < 0.01, examined by a two-tailed t-test.

To elucidate the mechanism by which RSV induces the expression of NKG2DLs, glioblastoma cells treated with RSV underwent RNA-Seq analysis. The analysis identified 5,400 differentially expressed genes (DEGs), with approximately 77% being upregulated, as illustrated by the volcano plot (**Figure 7A**, **Supplementary Figure 2A**). The violin plot demonstrated a relatively uniform data distribution across groups, confirming its suitability for subsequent functional analysis (**Supplementary Figure 2B**). Consistent with our previous findings, RNA-Seq revealed that RSV treatment significantly upregulated the mRNA levels of NKG2DLs, including ULBP2, ULBP3, ULBP5, and MICALL2, as further validated by qPCR (**Figures 7B, C**). These results suggest that RSV treatment increases NKG2DLs expression on glioblastoma cell surfaces. GO enrichment analysis indicated significant enrichment of DEGs in biological processes such as intracellular signal transduction, regulation of response to stimuli, and cellular component organization or biogenesis (**Supplementary Figure 2C**). Critically, KEGG analysis revealed significant enrichment in pathways that might facilitate the upregulation of NKG2DLs expression, including p53 signaling, Foxo signaling, MAPK/ERK signaling, PI3K-Akt signaling, and NF-κB signaling, as shown by GSEA analysis (**Figures 7D, E**). Molecular docking showed that RSV might bind to the p53 protein, with a binding energy of ≤-5 kcal/mol (**Figure 7F**). Western blot analysis further revealed that RSV treatment significantly enhanced the phosphorylated levels of p53 in glioblastoma cell lines (**Figures 7G, H**). Subsequently, we investigated whether p53 signaling was indispensable for the upregulation of NKG2DLs expression on glioblastoma cells. The results indicated that treatment with Pifithrin-α, an inhibitor of p53 signaling, markedly impeded the RSV-induced upregulation of NKG2DLs expression on glioblastoma cells (**Figure 7I**). Taken together, these findings indicate that RSV may enhance the expression of NKG2DLs on glioblastoma cells via p53 signaling pathway.

## 4 Discussion

CAR-T cells immunotherapy emerges great promise in the treatment of solid tumors, including glioblastoma. Despite several studies have suggested that CAR-T cell therapies targeting IL13Ra2, EGFRVIII, and HER2 have yielded encouraging results in clinical studies (31). Identifying suitable targets remains crucial for overcoming heterogeneous tumor antigen expression and immunosuppressive microenvironments (32). Our findings along with previous studies, indicate high NKG2DL expression in glioblastoma, particularly within the U251 cell line (17). Recent studies found that CAR-T cells targeting NKG2D exhibited anti-solid tumor efficacy and overcame immunosuppressive tumor microenvironment (TME) (33). Consistence with this phenomenon, our previous study suggested that NKG2D CAR-T cells displayed efficacy against glioblastoma 29. However, glioblastoma often evade immune recognition by downregulating or proteolytically shedding NKG2D ligands (19, 34). We therefore investigated the ability of this pharmacological adjuvant to upregulate NKG2D ligands expression on glioblastoma cells, thereby enhancing their sensitivity to NKG2D CAR-T cells and improving therapeutic efficacy.

RSV is a bioactive polyphenolic compound that enhances the NKG2D ligands expression on several cancer cells, including breast cancer cells, leukemia cells and leukemia stem cells-like, thereby increasing their susceptibility to natural killer cell-mediated cytotoxicity (35–37). Nevertheless, whether RSV upregulates the expression of NKG2D ligands on glioblastoma cells, remains unknown. Our investigation found that RSV-induced NKG2DL upregulation expression at the mRNA and protein levels enhanced antigen-specific recognition and cytotoxicity of NKG2D CAR-T cells to target cells. Specifically, the ULBP2/5/6 expression were increased on all cell lines, among of which U251 cells further upregulated. Critically, the NKG2DLs expression was higher on U251 cells compared with other glioblastoma cell lines. These findings were in accordance with our previous study showing that VPA treatment upregulated NKG2DLs expression on glioblastoma cells (29). Hence, using the U251 glioblastoma cell line, we found that RSV pretreatment significantly enhanced their sensitivity to NKG2D CAR-T cell-mediated killing. Consistent with enhanced target cell recognition via the NKG2D pathway, the cytotoxicity was accompanied by decreased secretion of IFN-γ and TNF-α, suggesting a reduced reliance on cytokine-driven effects. We hypothesize that RSV upregulates NKG2DLs expression, thereby sensitizing NKG2D CAR-T cells. According to previous literature, upon recognition of tumor antigens, these CAR-T cells undergo rapid polarization and exocytosis of Perforin and Granzyme-loaded cytotoxic granules, directly triggering apoptosis in target cells. Notably, this swift cytolytic process is not obligatorily coupled to the *de novo* synthesis of substantial quantities of IFN-γ or TNF-α (38). Our results also further suggested that RSV pretreatment significantly enhanced the expression of Granzyme. This may explain why RSV enhances NKG2D CAR-T cell cytotoxicity against gliomas while reducing IFN-γ and TNF-α secretion. However, these hypotheses require further validation in future studies. Consistent with no significant difference in IL-2 secretion, CAR-T cells proliferation was also no remarkable difference. This phenomenon prevents exhaustion caused by sustained TCR signaling, which may explain the downregulation of the exhaustion-associated markers TIM-3 and LAG-3. Overall, these results indicate that RSV upregulates NKG2D ligands on glioblastoma cells, thereby potentiating the direct cytotoxic function of NKG2D CAR-T cells.

Although high-dose CAR-T cell therapy is effective against solid tumors, it risks cytokine release syndrome (CRS) and on-target off-tumor effects (39). In a murine glioblastoma model, the combination of RSV and high-dose NKG2D CAR-T cells achieved superior tumor growth inhibition compared to CAR-T monotherapy, without inducing detectable toxicity in vital organs. Notably, RSV pretreatment enabled even low-dose CAR-T cells to inhibit tumor growth, suggesting that RSV lowers the effective CAR-T cell threshold required for efficacy. This advantage was further supported in an orthotopic glioma model, where the combination therapy inhibited tumor progression with minimal toxicity. To establish the generality of our orthotopic glioma model findings, independent validation across additional cell lines—coupled with direct visualization of CAR-T cell trafficking and tumoral infiltration—will be essential. Importantly, cytokine levels in peripheral blood and tumors were not significantly altered by RSV co-treatment, suggesting that the enhanced efficacy is not mediated by broad inflammatory activation. While no major organ injury was observed in this short-term study, the current model cannot fully assess the risk of on-target off-tumor effects, particularly given the broad ligand recognition profile of NKG2D CARs and the potential for RSV to upregulate these ligands on normal tissues. Future studies using normal-tissue co-culture systems or transgenic mouse models expressing human NKG2DLs are warranted to evaluate both the risk of on-target off-tumor effects and the long-term safety profile of this regimen, including histopathological assessment of immune organs, CRS monitoring, chronic inflammation, and potential delayed adverse effects.

A deep mechanistic understanding of the combinational therapeutic approach is essential to transform an initial discovery into an optimized and rational therapeutic strategy, paving the way for more effective solid tumor immunotherapies. Previous study has shown that MicroRNAs mediated gliomas evade immune surveillance by downregulating NKG2D ligands. Targeting these miRNAs with LNA inhibitors restores ligand expression and enhances susceptibility to NK cell lysis (40). In this study, we investigated the potential mechanism of RSV-induced expression of NKG2D ligands on glioblastoma cells. RNA-Seq revealed that Foxo signaling, MAPK signaling, p53 signaling, PI3K-Akt signaling and NF-κB signaling potential involved in RSV-induced expression of NKG2D ligands, among of which Gene Ratio of p53 signaling was higher. The molecular docking indicated that RSV could combine with p53 protein, as uncovered by the significantly phosphorylated upregulation of p53-induced by RSV treatment. Furthermore, inhibiting the p53 signaling reduced RSV-induced expression of NKG2D ligands on glioblastoma cells. Overall, RSV potentially induce NKG2D ligands expression via p53 signaling pathway. Future studies should further elucidate whether RSV induces NKG2D ligands expression potentially via other signaling pathways. Because RSV treatment activates multiple pathways associated with proliferation and survival (such as MAPK and PI3K), as well as pathways related to apoptosis and oxidative stress (such as FOXO), while also regulating inflammation-related pathways (NF-κB). This multi-target activity reflects both the pharmacologic pleiotropy of resveratrol and glioma biology—key considerations for deciphering combination therapy mechanisms.

Resveratrol upregulates NKG2DL through a mechanism distinct from established agents. Histone deacetylase inhibitors enhance NKG2DL transcription via chromatin remodeling and promoter acetylation (41); DNA-damaging agents trigger the ATM/ATR-dependent DNA damage response (42); and HSP90 inhibitors act indirectly by impairing client protein maturation (43). In contrast, our molecular docking and preliminary functional data suggest that resveratrol may operate through alternative routes potentially via p53-dependent transcriptional regulation. Supporting this notion, recent work has shown that resveratrol boosts NK cell−mediated killing by suppressing miR-17-5p and activating the MINK1/JNK/c-Jun axis to upregulate the ULBP2 (44). Beyond NKG2DL induction, RSV also exerts anti−inflammatory, antioxidant and direct anti−proliferative effects, which may remodel the tumor microenvironment to favor NK cell infiltration and function (44). Our previous study showed that sodium valproate combined with high-dose NKG2D CAR-T cells exerts antitumor activity. Here, we found that resveratrol combined with low-dose NKG2D CAR-T cells was sufficient to alleviate glioblastoma progression. Importantly, using an orthotopic intracranial glioblastoma model, we further elucidated the therapeutic advantages of this resveratrol-based combination strategy. However, it remains unclear whether resveratrol offers neuroprotective or antiepileptic benefits equivalent to those established for sodium valproate. Collectively, resveratrol is an attractive candidate for combination immunotherapy.

In conclusion, this study demonstrates that the natural compound resveratrol acts as a function adjuvant to potentiate NKG2D CAR−T cell therapy against glioblastoma. RSV may synergize with immunotherapy by triggering p53−mediated upregulation of NKG2D ligands on tumor cells. This combination yielded marked suppression of tumor growth *in vitro* and *in vivo*, without evident major organ toxicity. Our findings establish a rational combinatorial paradigm that addresses key barriers to solid−tumor immunotherapy, namely immune evasion.

## Data Availability

The original contributions presented in the study are included in the article/**Supplementary Material**. Further inquiries can be directed to the corresponding authors.
