# Supplementary material for: Combinatorial therapy with resveratrol sensitizes glioblastoma to NKG2D CAR-T cells
Source: Front Immunol. 2026 Jul 6;17:1831927. doi: 10.3389/fimmu.2026.1831927 (PMC13381691; doi:10.3389/fimmu.2026.1831927)
Supplement: Supplementary file 1 [file Table1.docx]

**Table S1. Primers for all selected genes were designed for real-time RT-PCR analysis in human samples**

| Accession No. | Primer name | Forward (‘5-3’) |
| --- | --- | --- |
| XM_047419377.1 | human ULBP2 | Forward: TGAAGGACACAGCAGTGGAT |
|  |  | Reverser: CATGGCCACAACCTTGTCAT |
| NM_024518.3 | human ULBP3 | Forward: GAAGGATAGCGGACTGACCA |
|  |  | Reverser: GGTTCCAGCCTCTTCTTCCT |
| NM_182924.4 | human MICALL2 | Forward: CTTGACCTACGTGTCCCAGT |
|  |  | Reverser: CTGGGCTGGAGATAGTGGAG |
| NM_001001788.4 | human ULBP5 | Forward: GTGGACAACGGTTCATCCTG |
|  |  | Reverser: CCATGCCCATCAAGAAGTCC |
| NM_002046.7 | human GAPDH | Forward: ACCCAGAAGACTGTGGATGG |
|  |  | Reverser:  TCAGCTCAGGGATGACCTTG |
