# Supplementary material for: Combinatorial therapy with resveratrol sensitizes glioblastoma to NKG2D CAR-T cells
Source: Front Immunol. 2026 Jul 6;17:1831927. doi: 10.3389/fimmu.2026.1831927 (PMC13381691; doi:10.3389/fimmu.2026.1831927)
Supplement: Supplementary file 2 [file DataSheet1.docx]

Supplementary Material

# Supplementary Data

#
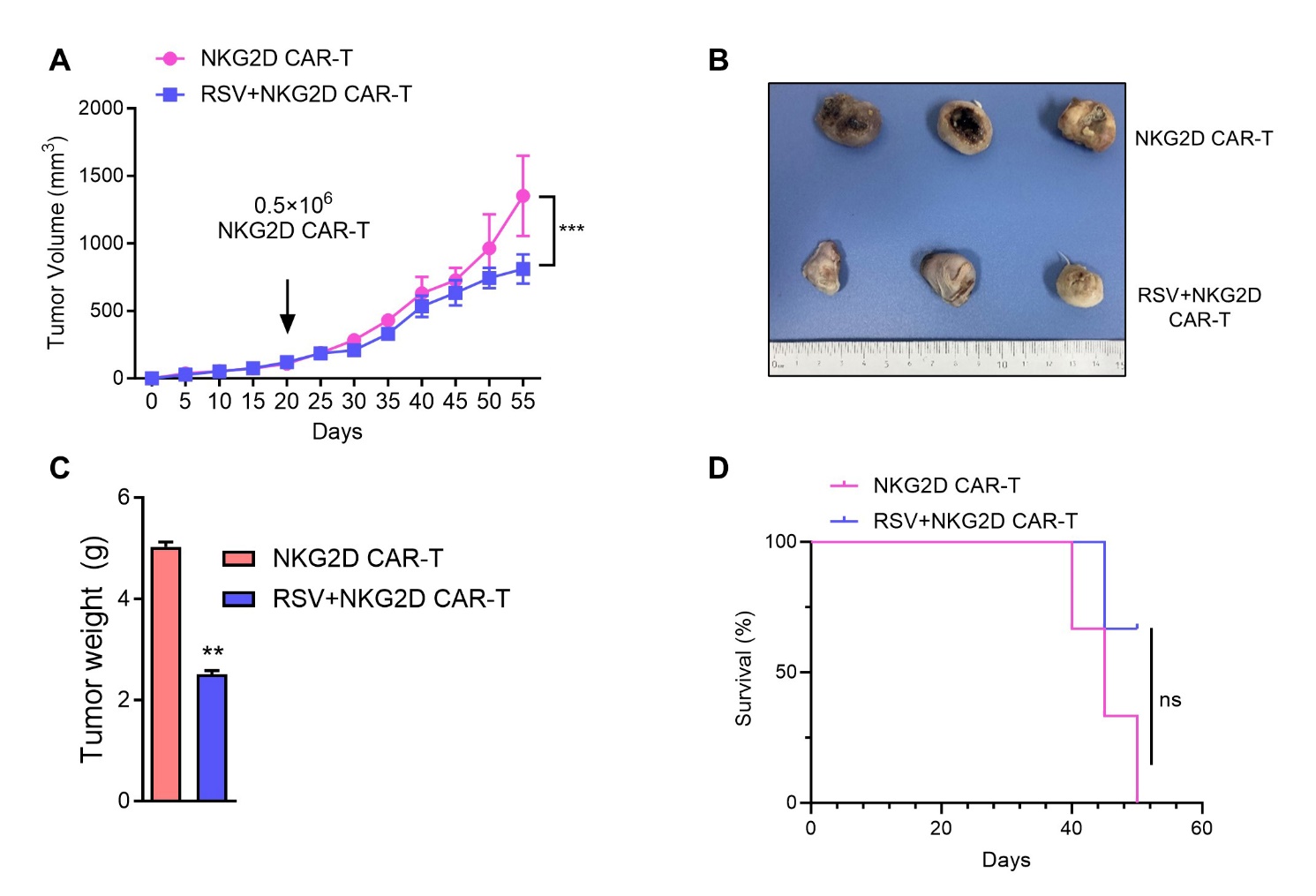


# Fig. S1 RSV combined with low-dose of NKG2D CAR-T cells treatment effectively inhibited glioblastoma growth in vivo.

# (A) Tumor volume measurement after tumor implantation in mice (n=3 mice per group). Arrows indicate the timing of intravenous infusion of engineered T cells. (B, C) Representative images of the excised tumor and tumor weight data were collected at the endpoint. (D) Kaplan-Meier survival curves of mice were recorded. All the experiments were repeated for three independent times. **: *p*<0.01, ***: *p*< 0.001, ns: no significance, examined by a two-tailed Student’s t test.

#
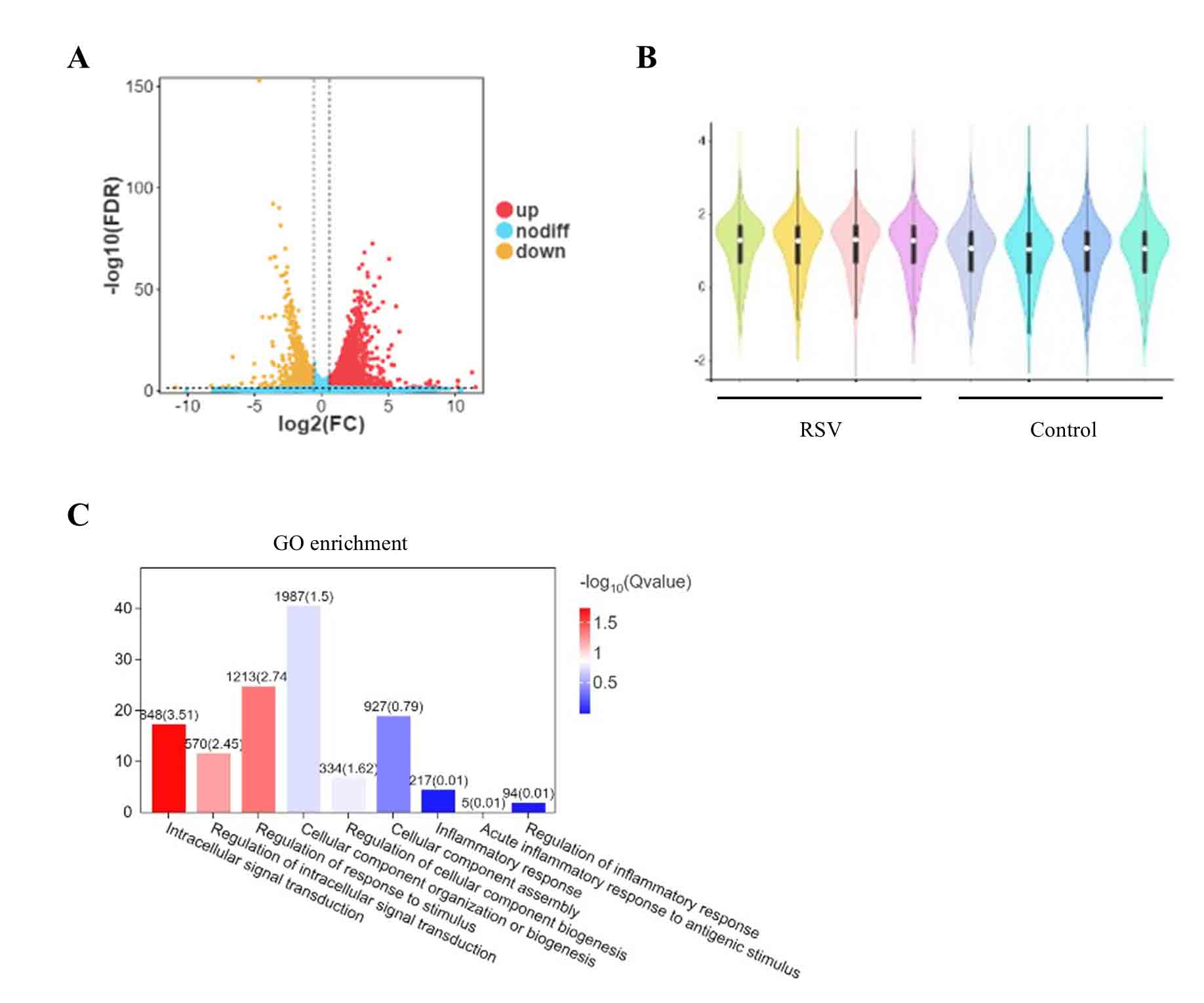


# Fig. S2 Biological processes were enriched by Go enrichment.

# (A) The upregulated and downregulated of gene were showed by heatmap (B) The distribution of expressed genes was visualized by Violin Diagram. (C) Go enrichment displayed biological processes upon RSV treatment.
